# Classification, Structure–Activity Relationship, and Anti‐Osteoarthritis Mechanism of Natural Flavonoids

**DOI:** 10.1002/cbdv.71719

**Published:** 2026-09-15

**Authors:** Haibin Wang, Shuqi Zheng, Chunlei Hua, Wanze Feng, Zengxia Liu, Yanhong Sun, Minhui Li

**Affiliations:** ^1^ Inner Mongolia Medical University Hohhot China; ^2^ Inner Mongolia Autonomous Region Hospital of Traditional Chinese Medicine Hohhot China

**Keywords:** cartilage protection, flavonoids, mechanism of action, natural products, osteoarthritis

## Abstract

Osteoarthritis is a chronic degenerative joint disease. Naturally derived flavonoids have become a hot spot in the field of osteoarthritis drug research and development. This paper summarizes the classification and chemical structure characteristics of flavonoids and the mechanism of anti‐osteoarthritis. The key structure–activity relationship (SAR) found that the hydroxyl group of o‐diphenol/o‐triphenol on the B‐ring can scavenge free radicals and chelate pro‐oxidant metal ions, which directly determines the antioxidant strength; the C2═C3 double bond on the C‐ring is conjugated with the 4‐carbonyl group to form a rigid planar structure, which can enhance the anti‐inflammatory activity; isoflavones linked to C‐3 in the B‐ring can regulate cartilage metabolism through estrogen receptors. The C‐3 hydroxyl group in flavonols further enhances the free radical scavenging efficiency through intramolecular hydrogen bonds. Although there is sufficient preclinical evidence, the oral bioavailability of most flavonoids is extremely low and there is a lack of high‐quality clinical studies and long‐term randomized controlled trials. Future research should optimize the structure of compounds based on SAR, and carry out rigorous clinical trials to promote flavonoids from laboratory to clinical application.

## Graphical abstract

Natural product flavonoids have a multi‐target therapeutic intervention effect on osteoarthritis. They promote autophagy and cartilage synthesis while alleviating oxidative stress, inhibiting inflammation, and inhibiting matrix degradation. By restoring OPG/RANKL balance and regulating bone metabolism, these compounds synergistically delay the progression of osteoarthritis and promote cartilage repair.

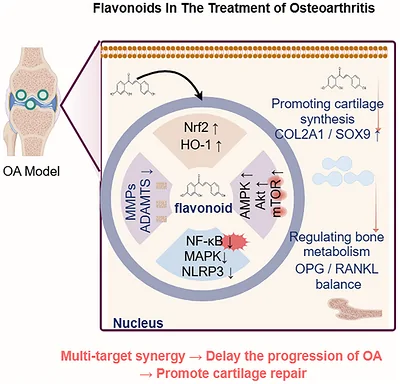

**Abbreviations**
ACLT: anterior cruciate ligament transection
ADAMTS: a disintegrin and metalloproteinase with thrombospondin motifs
ADME: absorption, distribution, metabolism, excretion
AGEs: advanced glycation end products
Akt: protein kinase B
AMPK: AMP‐activated protein kinase
AP‐1: activator protein 1
ARE: antioxidant response element
Bax: Bcl‐2‐associated X protein
Bcl‐2: B‐cell lymphoma 2
BMSCs: bone marrow mesenchymal stem cells
CAV‐1: caveolin‐1
COL2A1: collagen type II alpha 1 chain
COX‐2: cyclooxygenase‐2
DMM: destabilization of the medial meniscus
ECM: extracellular matrix
EGCG: epigallocatechin‐3‐gallate
EIF6: eukaryotic translation initiation factor 6
ER: estrogen receptor
ERE: estrogen response element
ERK: extracellular signal‐regulated kinase
FPN: ferroportin
GPX4: glutathione peroxidase 4
GSH: glutathione
HIF‐2α: hypoxia‐inducible factor‐2α
HO‐1: Heme oxygenase‐1
IL‐1β: interleukin‐1β
IL‐6: interleukin‐6
iNOS: inducible nitric oxide synthase
JAK2: janus kinase 2
JNK: c‐jun N‐terminal kinase
LPS: lipopolysaccharide
MAPK: mitogen‐activated protein kinase
MIA: monosodium iodoacetate
MMP: matrix metalloproteinase
MMP‐13: matrix metalloproteinase 13
mTOR: mammalian target of rapamycin
mTORC1: mammalian target of rapamycin complex 1
MyD88: myeloid differentiation primary response 88
NF‐κB: nuclear factor kappa‐B
NLRP3: NLR family pyrin domain containing 3
NO: nitric oxide
NOX4: NADPH oxidase 4
NQO‐1: NAD(P)H:quinone oxidoreductase 1
Nrf2: nuclear factor erythroid 2‐related factor 2
NSAIDs: nonsteroidal anti‐inflammatory drugs
OA: osteoarthritis
OPG: osteoprotegerin
P2X7: P2X purinoceptor 7
PAs: proanthocyanidins
PF127: pluronic F127
PGE2: prostaglandin E2
PI3K: phosphoinositide 3‐kinase
RANKL: receptor activator of nuclear factor‑κB ligand
ROS: reactive oxygen species
SASP: senescence‐associated secretory phenotype
SIRT1: sirtuin 1
SIRT6: sirtuin 6
SLC7A11: solute carrier family 7 member 11
Smad: mothers against decapentaplegic homolog
SOD: superoxide dismutase
SOX9: SRY‐box transcription factor 9
STAT1/2/3: signal transducer and activator of transcription 1/2/3
TfR1: transferrin receptor 1
TGF‐β: transforming growth factor‐β
TLR4: toll‐like receptor 4
TNF‐α: tumor necrosis factor‐α
TRPV1: transient receptor potential vanilloid 1
TSC1: tuberous sclerosis complex1
ULK1: Unc‐51 like autophagy activating kinase 1
Wnt: wingless‐related integration site

## 1 Introduction

Osteoarthritis (OA) is a chronic degenerative joint disease with progressive loss of articular cartilage, synovitis, and pathological remodeling of subchondral bone [1]. Globally, the incidence and prevalence of OA continue to rise, which not only brings a heavy burden to patients, but also poses a serious challenge to the public health system [2]. The pathological process involves the release of pro‐inflammatory factors, accelerated degradation of extracellular matrix and abnormal increase of chondrocyte apoptosis [3], which eventually leads to the destruction of joint structure and loss of function. The clinical manifestations are pain, swelling, and limited activity [4]. The initial symptoms of the disease are mostly intermittent joint pain and morning stiffness. With progression of the disease, joint deformity and loss of function may occur, significantly impacting the quality of life of the patients.

At present, the treatment methods recommended by clinical guidelines at home and abroad are mainly symptomatic treatment, including nonsteroidal anti‐inflammatory drugs and intra‐articular injection of glucocorticoids [5]. However, these methods can only temporarily relieve symptoms and cannot delay or reverse the process of cartilage degeneration [6], long‐term use may also cause gastrointestinal and cardiovascular adverse reactions [1]. Therefore, the development of new therapeutic drugs with both disease‐modifying ability and good safety has become an urgent need in the field of OA research [7, 8].

In recent years, the potential and mechanism of natural product extracts in the treatment of OA have received extensive attention. Among the lead compounds derived from many natural products, flavonoids have shown particularly prominent potential in OA intervention studies [9]. This advantage is not accidental, but stems from its unique chemical skeleton and structural diversity. From the perspective of chemical taxonomy, flavonoids share the basic skeleton of C6─C3─C6, but they can be further divided into flavonoids, flavonols, dihydroflavones, isoflavones, flavanols, anthocyanins, and chalcones according to the degree of oxidation, saturation, and B‐ring connection position of C‐ring (oxygen‐containing heterocycles) [10, 11]. This structural “finite skeleton, infinite modification” feature gives flavonoids unique advantages over other natural product categories, such as structural modifiability and druggability optimization space, multi‐channel synergy, good safety, and less resistance [12].

In recent years, a large number of cell and animal model studies have confirmed that a variety of flavonoids (luteolin, quercetin, kaempferol, EGCG, genistein, etc.) have a definite cartilage protective effect in OA [13]. Some compounds (quercetin and EGCG) have entered the clinical trial stage, and the safety and efficacy of improving pain and joint function in OA patients have been preliminarily verified. However, most of the existing reviews focus on the mechanism summary of a single compound or a single category [14], and lack systematic analysis from chemical structure classification to structure–activity relationship (SAR) rules to mechanism of action and clinical transformation.

The purpose of this paper is to systematically review the pharmacological mechanism of flavonoids in the prevention and treatment of OA, to deeply explain its multi‐target therapeutic principle, and to explore the SAR between its chemical structure (including parent nucleus type, functional group modification and stereochemical characteristics) and biological activity, and to clarify the molecular basis of existing activity differences, in order to provide a theoretical basis for the development of new OA treatment drugs.

### 1.1 Literature Retrieval Strategy

In order to ensure the transparency and repeatability of this review, we systematically searched the relevant literature published up to July 2026. The electronic databases included PubMed, Web of Science, Scopus, and CNKI. The search strategy combines keywords and free words related to flavonoids and OA. The main search terms include “flavonoids,” “natural products,” “osteoarthritis,” “cartilage protection,” “inflammation,” “signaling pathway,” and use Boolean logic operators (AND, OR) for combination. In addition, we also manually screened the list of references and related review articles included in the study to supplement the search for qualified literature that may be missing.

The inclusion criteria of the literature were as follows: (1) To study the mechanism of flavonoids in OA; (2) in vitro, in vivo, or clinical studies; (3) published in English or Chinese. Exclusion criteria: (1) studies unrelated to OA or flavonoids; (2) conference abstracts, editorials or case reports lacking original data; (3) The data are not enough for effective analysis. Although this article is not a formal meta‐analysis and does not strictly follow the PRISMA 2020 statement list, we have followed the PRISMA principles to the maximum extent possible during the search and screening process to improve methodological rigor and ensure full coverage of existing evidence.

## 2 The Pathogenesis of OA

OA is a joint disease affected by many factors, including mechanical, metabolic, inflammatory and hereditary interactions. Pathology of OA is associated with the slow destruction of the articular cartilage, synovitis and abnormalities of subchondral bone. These types of processes are triggered by a sequence of cellular and molecular events that eventually lead to the loss of joint functions and the occurrence of pain.

### 2.1 Cartilage Degradation and Synovial Inflammation

Articular cartilage is maintained by chondrocytes, which regulate the balance between anabolism and catabolism. In OA, this balance is broken due to mechanical stress, aging, or inflammatory stimulation. Phenotypic changes in chondrocytes result in increased production of matrix‐degrading enzymes, such as matrix metalloproteinases and depolymerizing protein‐like metalloproteinases (especially MMP‐13 and ADAMTS‐5) containing platelet‐binding protein motifs, which degrade type II collagen and aggrecan, respectively. Simultaneously, the formation of extracellular matrix elements (type II collagen and proteoglycans) decreases, resulting in the disruption of the cartilage integrity, and causing the development of OA [15, 16].

At present, it is generally believed that synovitis is one of the important causes of the occurrence and development of OA. Synovial tissues that are inflamed produce pro‐inflammatory cytokines, including interleukin‐1β, tumor necrosis factor‐α and interleukin‐6, which remain to cause cartilage loss, recruit immune cells, and so on. Pro‐inflammatory M1 phenotype is formed by macrophages of the synovium, which release cytokines and reactive oxygen species, which further worsens the state of the joint. Such an inflammatory milieu also supports angiogenesis and sensory nerve growth, both of which may induce pain and contribute to the advancement of OA [17, 18].

The above main driving factors are the abnormal activation of NF‐kβ and MAPKs (p38, JNK, ERK), resulting in over‐expression of pro‐inflammatory cytokines and catabolic enzymes, thereby degrading type II collagen and aggrecan, and destroying cartilage ECM [19]. At the same time, the PI3K/Akt/mTOR pathway that regulates cell survival and autophagy is often dysfunctional, further exacerbating ECM destruction and chondrocyte dysfunction. Flavonoids can effectively inhibit the activation of NF‐kβ and MAPK, and directly antagonize the above inflammatory and catabolic cascades [20]. In addition, Wnt/β‐catenin and OPG/RANKL axis are the key regulatory pathways of bone remodeling. Some flavonoids can also regulate these pathways, slow down the changes of subchondral bone and maintain the overall structural integrity of joints [21].

### 2.2 Oxidative Stress and Autophagy Disorders

The oxidative stress is one of the main factors in the pathogenesis of OA [22]. Reactive oxygen species are increased in chondrocytes and synovial cells, causing mitochondrial dysfunction, lipid peroxidation, and DNA damage, which contribute to cell senescence and apoptosis. The Nrf2 pathway is the main regulator of cellular antioxidant response. Nrf2 signaling in OA is often impaired and aggravates disease progression. Many flavonoids, especially catechol‐containing compounds, are potent activators of the Nrf2/HO‐1 pathway, which can directly scavenge ROS and indirectly enhance endogenous antioxidant defenses and restore redox homeostasis in chondrocytes [23].

The aged cells also display aging‐related secretory phenotypes, secrete pro‐inflammatory agents, enhance tissue destruction and inflammation, and hasten the development of OA [24]. Autophagy is a mechanism of cell survival that eliminates damaged organelles and proteins. OA chondrocyte autophagy is disrupted because of age and stress resulting in higher levels of apoptosis and extracellular matrix breakdown [25]. Flavonoids (icariin, genistein) can activate the PI3K/Akt pathway, promote chondrocyte survival and induce protective autophagy, while inhibiting apoptosis through the mitochondrial pathway.

**FIGURE 1:**
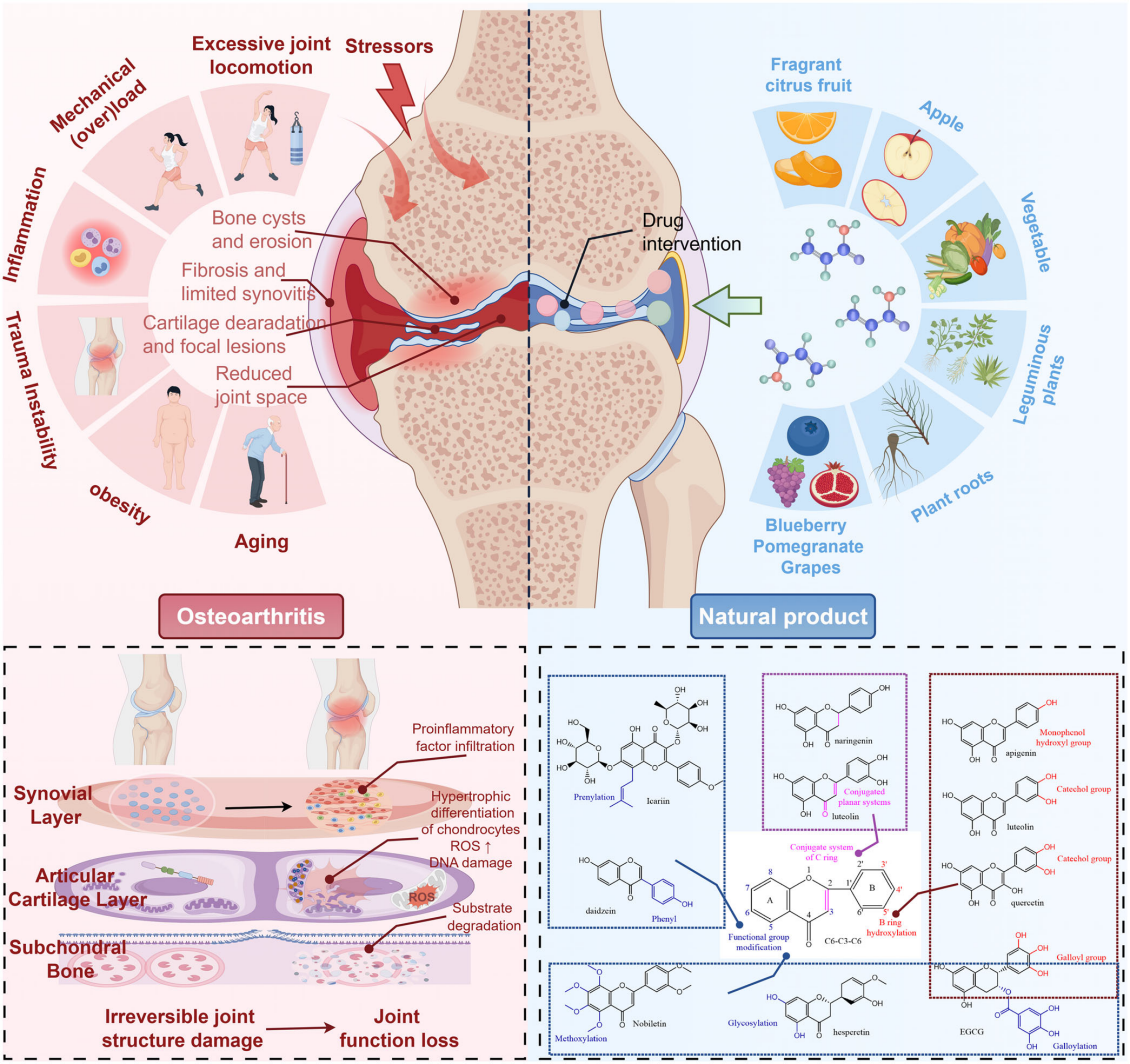
Schematic diagram of the multi‐target mechanisms of flavonoids in osteoarthritis. (By Figdraw.).

To sum up, the pathogenesis of OA is a complicated web of cell dysfunction, inflammatory signaling, oxidative stress, and metabolism disorder. The linked pathways offer numerous opportunities to intervene therapeutically, and the flavonoids with wide biological action are considered as one of the most promising candidates in the treatment of OA [26] (Figure 1).

## 3 Classification of Flavonoids and Mechanism of Anti‐Osteoarthritis

**FIGURE 2:**
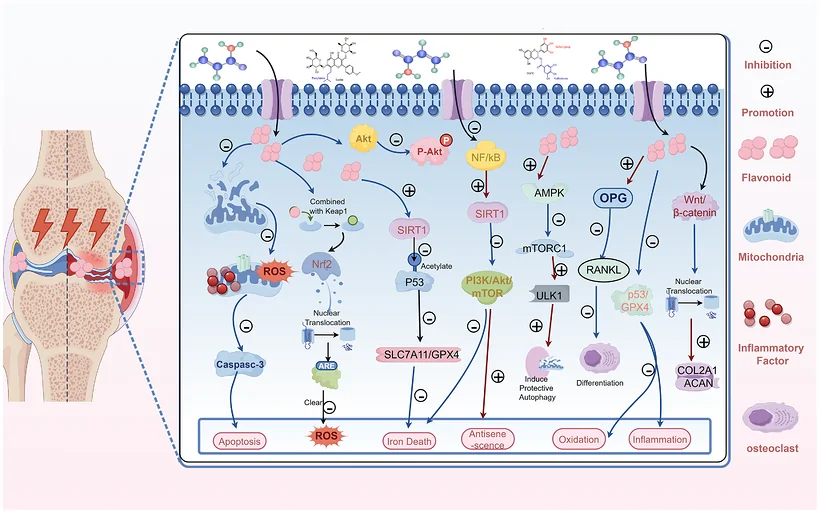
Overview of anti‐osteoarthritis signaling pathway of flavonoids. (By Figdraw.).

Flavonoids are polyphenolic secondary metabolites widely found in fruits, vegetables, tea, wine, and medicinal plants. A large number of cells, animals, and preliminary clinical studies have shown that they have anti‐arthritis potential. According to its chemical structure, it can be divided into several subclasses, including flavonoids, flavonols, flavanones, isoflavones, flavanols, anthocyanins, and other subclasses. The biological activity of these compounds is deeply affected by their chemical structure, which is mainly reflected in the fact that the parent core structure determines the basic skeleton and electron cloud distribution. The modification of functional groups on the B‐, A‐, and C‐rings (such as the number, type, and position of hydroxyl, methoxy, and sugar groups) significantly affected their antioxidant capacity, affinity to signal proteins, and hydrophilic lipophilicity; the stereochemical characteristics of the C‐ring (such as double bond configuration and chiral center) affect the spatial conformation of the molecule, which in turn determines the specificity of its binding to the receptor or enzyme. The following will be combined with representative flavonoids to explore its mechanism of action in the treatment of OA (Figure 2).

### 3.1 Flavonoids

The general structure of flavonoids is 2‐phenylchromone, and it has a double bond between the 2‐3 positions of the C‐ring and a carbonyl at the 4th position. The planar shape allows it to interact with the binding sites of some enzymes or receptors.

#### 3.1.1 Luteolin

Luteolin is a natural flavonoid compound widely found in celery, broccoli, apple, honeysuckle, and other fruits and vegetables and Chinese herbal medicines. It has strong antioxidant and anti‐inflammatory properties. Its structure is 5,7,3',4'‐tetrahydroxyflavone. The catechol hydroxyl group (3',4'‐dihydroxy) on the B‐ring is the key structural motif of its strong antioxidant activity, which can effectively chelate metal ions and scavenge free radicals. A large number of studies have shown that luteolin can effectively reduce the production of key inflammatory factors such as IL‐1β, TNF‐α, and IL‐6 by inhibiting the activation of NF‐κB signaling pathway and regulating the MAPK pathway [27]. In a variety of models, it has been shown to inhibit the expression of inflammatory mediators and proteolytic enzymes and reduce collagen destruction. Concurrently, it has the ability to reduce the induction of matrix metalloproteinases (MMP‐1, MMP‐3, and MMP‐13) by IL‐1β, and enhance the production of type II collagen and proteoglycan in chondrocytes, which would help to preserve the extracellular matrix [28]. In addition, luteolin can directly remove reactive oxygen species, reduce oxidative stress damage by activating AMPK‐Nrf2 signaling [29], and maintain intracellular homeostasis by activating chondrocyte autophagy flow to delay OA progression [30].

#### 3.1.2 Apigenin

As a natural flavonoid compound, apigenin shows a multi‐target protective effect in the treatment of OA. Compared with luteolin, apigenin has only one 4'‐hydroxyl on the B‐ring, so its antioxidant capacity is relatively weak, but it shows unique biological effects. In addition to conventional inhibition of NF‐κB and MAPK pathways [31, 32], the unique mechanism of apigenin is that it can target EIF6 to alleviate chondrocyte senescence and block HIF‐2α to inhibit the activity of MMP‐3 and MMP‐13 [33], thereby synergistically delaying cartilage degradation [34]. In addition, it can also induce macrophages to differentiate into anti‐inflammatory M2 phenotype, highlighting its role in regulating the immune microenvironment of joints [35].

Although luteolin and apigenin have shown good efficacy in vitro and in vivo experiments, these two flavonoids are mainly present in the form of glycosides in natural sources, and their absorption is hindered by poor water solubility, strong intestinal metabolism, and rapid liver glucuronidation/sulfation, resulting in extremely low systemic bioavailability [36]. In most rodent OA models, luteolin and apigenin are usually administered intraperitoneally or orally at a dose of 10 to 100 mg/kg; if it is converted into human equivalent dose, it is often much higher than the level that can be achieved through dietary intake or conventional oral supplements [37]. Pharmacokinetic studies have shown that the peak concentration of free luteolin or apigenin in human blood rarely reaches the micromolar range, while the effective concentration based on cell experiments is usually at a low micromolar level [27]. Therefore, it is still uncertain whether the current in vivo administration regimen can be directly applied to clinical practice.

#### 3.1.3 Baicalein

Baicalein is a natural flavonoid extracted from the dried roots of *Scutellaria baicalensis*. It has a long history of application in traditional medicine. Its unique A‐ring 6‐hydroxyl group plays an important role in its pharmacological activity. Its anti‐OA mechanism mainly includes effective inhibition of NF‐κB signaling pathway and phosphorylation of MAPK pathways such as p38 and JNK, thereby synergistically inhibiting the expression of inflammatory factor genes and the production of inflammatory mediators such as PGE2 and NO [38, 39]. Studies have also shown that baicalein can inhibit the differentiation and activity of osteoclasts, slow down the abnormal absorption of subchondral bone, and is of great significance for maintaining the overall structural integrity of the joint [40, 41].

#### 3.1.4 Other Flavonoids

**FIGURE 3:**
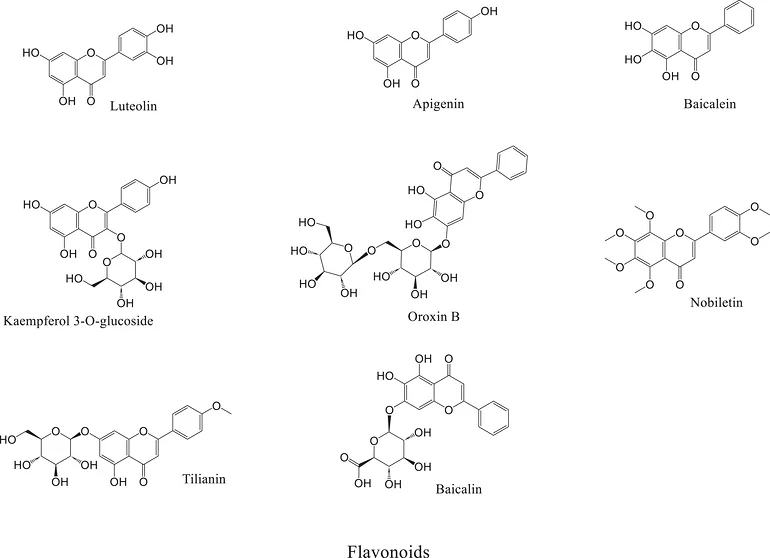
Chemical structures of representative Flavonoids. (All chemical structures were drawn using ChemDraw (Version 22.0, CambridgeSoft).

Besides the aforementioned few flavonoids, numerous other flavonoids and their derivatives have also been shown to be potentially active in OA treatment. For example, astragaloside extracted from *Astragalus membranaceus* can reduce the production of inflammatory mediators by inhibiting NF‐κB and MAPK pathways, and shows protective effects in IL‐1β‐induced chondrocytes and ACLT rat models [42]. Oroxylin B can down‐regulate the expression of MMP‐3, MMP‐13, and ADAMTS5, and inhibit COX‐2, thereby reducing cartilage degradation [43]. Nobiletin inhibits inflammation and cartilage degeneration by antagonizing NF‐κB and PI3K/Akt pathways. Baicalin can inhibit macrophage M1 polarization and promote M2 phenotype, and regulate joint immune microenvironment. By inhibiting the TLR4/NF‐κB pathway, tilianin reduces inflammation, oxidative stress and apoptosis, and helps maintain extracellular matrix homeostasis [44]. In addition, morin extracted from *Phellinus igniarius* can inhibit the p38 MAPK pathway, reduce the production of inflammatory mediators, and reduce chondrocyte apoptosis and inflammatory response (Figure 3). In summary, by comparing the structures of these compounds, it can be found that the difference in activity is closely related to the hydroxylation mode and the degree of methoxylation. For example, polymethoxyflavones (e.g., nobiletin) may be more suitable for oral administration due to their lipid‐enhancing solubility of the methoxy group, which usually has better cell membrane permeability and metabolic stability.

### 3.2 Flavonols

The difference between flavonols and flavonoids is that the hydroxyl substitution at the 3‐position of the C‐ring is present. This structural characteristic also boosts its antioxidant potential and is able to form additional intramolecular hydrogen bonds.

#### 3.2.1 Quercetin

Quercetin is one of the most widely distributed flavonols in nature. It is found in onion, apple, and tea. The coexistence of o‐diphenol hydroxyl group in B‐ring and 3‐OH in C‐ring in quercetin structure forms an efficient antioxidant combination. In the OA chondrocyte model, quercetin treatment can significantly reduce the level of inflammatory factors, reduce the production of MMPs, and promote the synthesis of type II collagen and proteoglycan [45]; at the same time, Nrf2/HO‐1 and AMPK/Nrf2/Gpx4 pathways are activated to enhance the antioxidant defense of cells and resist ferroptosis [13, 46]. Animal experiments have confirmed that oral or intra‐articular administration of quercetin can reduce articular cartilage injury, synovial inflammation, and pain‐related behaviors [47, 48].

Although quercetin has shown significant cartilage protection and anti‐inflammatory effects in in vitro experiments and animal models, it's extremely low oral bioavailability has become a major obstacle to clinical application. After oral administration, quercetin undergoes extensive phase II metabolic processes in the intestine and liver, most of which are converted into glucuronide and sulfate conjugates and are rapidly removed, resulting in a systemic bioavailability of less than 5% in most pharmacokinetic studies [49]. Therefore, the concentration of free aglycones in joint tissue is usually much lower than the level required to exert its proven pharmacological effects [50]. This gap requires exploring a variety of preparation strategies. For example, nano‐preparations are expected to improve the water solubility of quercetin, resist gastrointestinal degradation, and improve its accumulation in articular cartilage by enhancing permeability and retention effects. In addition, structural modifications (the synthesis of quercetin phosphate prodrugs or methylated derivatives) provide another way to improve oral bioavailability while maintaining biological activity [51]. Although these methods are still mainly in the preclinical stage, they have pointed out the direction for overcoming bioavailability barriers and promoting quercetin to become a feasible drug for OA treatment.

#### 3.2.2 Kaempferol

Kaempferol is commonly found in dark green vegetables and beverages such as spinach and green tea. Compared with quercetin, its B‐ring lacks 3'‐hydroxyl, and its antioxidant properties are weaker than quercetin, but its inhibition of certain kinases may be more selective. In a recent meta‐analysis summary [52], kaempferol significantly reduced the expression of inflammatory factors and MMPs by inhibiting multiple signaling pathways such as NF‐κB, MAPK, and PI3K/Akt/mTOR, and effectively reduced cartilage destruction and osteophyte formation in multiple animal models [53]. In the IL‐1β‐induced chondrocyte model, kaempferol enhances cell antioxidant capacity by activating the Nrf2/HO‐1 pathway and inhibits chondrocyte pyroptosis and senescence. In addition, kaempferol can inhibit inflammation and extracellular matrix degradation from the epigenetic level by regulating noncoding RNA networks such as the XIST/miR‐130a/STAT3 axis [54]. It is also possible that it will stimulate the osteogenic differentiation of bone marrow mesenchymal stem cells through the inhibition of CAV‐1, which plays a role in the remodeling of subchondral bone [55]. These effects are synergistic with mechanisms such as regulating the PI3K/Akt/mTOR pathway and inhibiting pyroptosis, which effectively alleviates the progression of OA.

#### 3.2.3 Isorhamnetin

Isorhamnetin is a natural flavonol compound widely found in sea buckthorn, wolfberry, ginkgo, and other plants. It is a 3'‐methoxylated derivative of quercetin. The introduction of methoxy increases the lipophilicity of the molecule, which may improve its transmembrane transport and interaction with intracellular targets, so that it has enhanced bioavailability and antioxidant activity. In the study of OA, isorhamnetin showed significant cartilage protection and anti‐inflammatory effects [56]. Isorhamnetin can significantly inhibit the inflammatory response of rat chondrocytes induced by IL‐1β, inhibit the excessive activation of PI3K/Akt/mTOR signaling pathway, induce protective autophagy of chondrocytes, maintain cell homeostasis, and delay cartilage degeneration. It can effectively reduce the expression of inflammatory mediators such as IL‐1β, TNF‐α, COX‐2, and inhibit the activity of matrix degradation enzymes such as MMP‐3 and MMP‐13, thereby reducing the destruction of extracellular matrix. In addition, isorhamnetin has strong free radical scavenging ability, which can reduce chondrocyte damage caused by oxidative stress and inhibit inflammation‐induced apoptosis. At present, isorhamnetin has shown good therapeutic potential in a variety of cell and animal OA models, especially in the regulation of autophagy and inflammation balance, which provides a theoretical basis for its further development of OA treatment drugs [57].

#### 3.2.4 Other Flavonol Compounds

**FIGURE 4:**
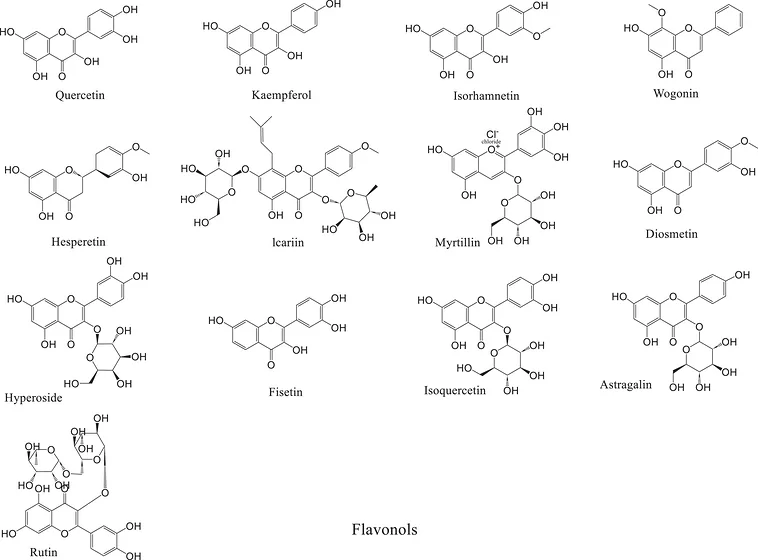
Chemical structures of representative flavonols. (All chemical structures were drawn using ChemDraw (Version 22.0, CambridgeSoft).

In addition to the aforementioned compounds, other flavonol compounds also show various protective effects in the treatment of OA. For example, wogonin can inhibit AP‐1 and JAK2/STAT1/2 pathways, down‐regulate MMP‐13 expression, and reduce cartilage extracellular matrix degradation [58]; hesperetin has antioxidant and anti‐inflammatory properties because it activates the Nrf2/HO‐1 pathway and blocks the NF‐κB signaling [59]; icariin can inhibit the NF‐κB/HIF‐2α pathway and reduce the expression of MMP‐9 and ADAMTS‐5 [60]; myrtillin could partially block the PI3K/Akt/NF‐κB and MAPK pathways and inhibit inflammation and matrix degradation. In addition, diosmetin promoted the transformation of macrophages from M1 to M2 by inhibiting the PI3K/AKT signaling pathway, and improved the inflammatory environment of joints [61]; hyperoside exerts anti‐inflammatory, antioxidant, and antiapoptotic effects by inhibiting the PI3K/AKT/NF‐κB pathway. Fisetin inhibits chondrocyte senescence by targeting the SIRT6/Nrf2/HO‐1 pathway. Isoquercetin exerts antiaging effects by regulating the NOX4/Nrf2 redox balance [62]; astragalin reduced cartilage degradation by inhibiting JNK/p38 MAPK pathway and reducing the expression of inflammatory factors and MMP3. Both isoquercitrin and rutin inhibit the inflammatory response and relieve the symptoms of OA by regulating the inflammatory pathway. The above compounds and their mechanism of action were verified in related OA models. It is interesting to note that icariin is an 8‐prenylflavonol. Prenylation has been shown to greatly improve the lipophilicity of the molecule as well as its affinity toward some targets, which makes it exceptional when it comes to the regulation of bone metabolism. Glycosylation (hyperoside, isoquercitrin, rutin, and astragaloside) tends to occur at the 3‐OH position. Glycosylation also contributes a lot to water solubility of the compound, and reduces membrane permeability and the ability to directly bind intracellular targets. Its action is frequently determined by the aglycone deglycosylation products of the intestinal flora (Figure 4).

The overall antioxidant capacity of flavonols is stronger than that of flavonoids, and quercetin is the compound with the most sufficient preclinical evidence [11]. However, the oral bioavailability of quercetin is extremely low (<5%), and it mainly exists in the form of metabolites in the body, which seriously limits its clinical transformation. Although the antioxidant activity of kaempferol is slightly weaker, its unique role in epigenetic regulation and osteogenic differentiation suggests that stronger antioxidant activity is not the only goal, and flavonols with complementary mechanisms should be selected for different pathological features of OA [63]. The methoxy modification of isorhamnetin provides a method for improving oral absorption, but it's in vivo activity data are still limited [64].

### 3.3 Flavanones

The feature of flavanones is that the 2‐3 positions of the C‐ring are single bonds, which are saturated, causing the C‐ring to not be coplanar and the entire molecule to be bent. The present conformational change has a major influence on the manner in which it binds to the target molecule.

#### 3.3.1 Hesperidin

Hesperidin is mainly present in the peel of citrus fruits. It is a 7‐O‐rutinoside of hesperetin (5,7,3'‐trihydroxy‐4'‐methoxyflavanone). The presence of glycosyl makes it highly water‐soluble, but the oral bioavailability is low. It down‐regulates the production of inflammatory mediators such as MMPs, PGE2, and NO by inhibiting NF‐κB and MAPK signaling pathways [59]. In the IL‐1β‐induced chondrocyte model, hesperidin can directly inhibit IL‐1β‐induced inflammatory response [65] and protect chondrocytes from oxidative damage through the PI3K/Akt pathway [66]. A key issue that is often overlooked is that hesperidin, as a glycoside, needs to be deglycosylated by the intestinal flora to release the active aglycone‐hesperetin, which is the form that truly exerts pharmacological effects [67]. The β‐glucosidase of the intestinal flora converts hesperidin into hesperetin, which is then absorbed and further metabolized. Significant individual differences in the composition of gut microbiota may lead to significant differences in the bioavailability and therapeutic effect of hesperidin [68]. These factors should be fully considered in the design of clinical research and individualized treatment.

#### 3.3.2 Naringenin

Naringenin is a natural flavonoid mainly found in citrus fruits. It is one of the main sources of citrus bitterness. Compared with hesperetin, its B‐ring lacks 3'‐hydroxyl and 4'‐hydroxyl is not methoxylated. It also has antioxidant, anti‐inflammatory, and antiapoptotic effects. It down‐regulates the expression of MMPs, IL‐6 and COX‐2 by inhibiting NF‐κB and MAPK pathways [69]. Importantly, naringenin can synergistically reduce inflammation and cell damage at the cellular level by activating the SIRT1‐mediated protective autophagy pathway and inhibiting the activation of NLRP3 inflammasome. In addition, in vivo studies have shown that naringenin can alleviate OA induced by anterior cruciate ligament transection or sodium iodoacetate in rats, and its mechanism is related to the regulation of TGF‐β/Smad3 signaling pathway and the improvement of subchondral bone sclerosis [70].

In order to improve the bioavailability and targeting of flavanone compounds such as hesperidin and naringenin, nanocarrier systems have become a research hotspot. For example, naringenin nanopreparations can significantly enhance their intra‐articular retention time and cartilage targeting, thereby showing stronger cartilage protection in rat OA models [71]. Similarly, liposome‐encapsulated hesperidin also showed enhanced anti‐inflammatory and cartilage repair effects [72], providing a new idea for breaking through the pharmacokinetic bottleneck of such compounds.

#### 3.3.3 Eriocitrin

**FIGURE 5:**
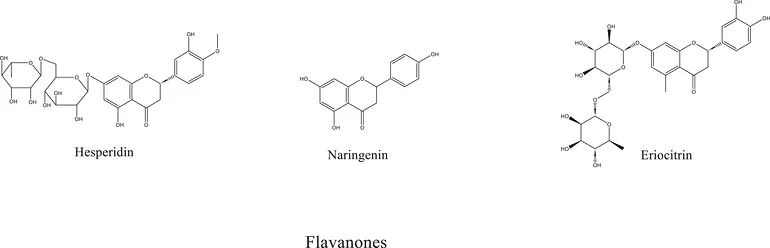
Chemical structures of representative flavanones. (All chemical structures were drawn using ChemDraw (Version 22.0, CambridgeSoft).

The amount of eriocitrin in lemon juice and lemon peel is especially large. It is an aglycone that shows a prepotential of OA after hydrolysis in vivo to produce the active eriodictyol. Luteolin and eriocitrin are identical in their B‐ring catechol hydroxyl, but their C‐ring is a saturated bond. This comparative analysis of both structure and activity is useful to clarify the role of C‐ring configuration. Its main action is to efficiently lower the concentration of major pro‐inflammatory mediators in the joint through the inhibition of NF‐κB and MAPK inflammatory signaling pathways; simultaneously, the Nrf2/HO‐1 antioxidant pathway is induced to increase the self‐defensive properties of the chondrocytes [73]. In regard to cartilage preservation, it is able to greatly suppress the actions of matrix degrading enzymes, decrease the loss of cartilage matrix, and stimulate the synthesis of collagen II stimulated by SOX9, and preserve the cartilage integrity and prevent the chondrocyte apoptosis (Figure 5).

In general, dihydroflavones lose their planarity due to C‐ring saturation, and their direct antioxidant capacity is usually weaker than that of corresponding flavonoids, but conformational changes may increase their selectivity to certain protein targets [74]. For example, the inhibitory effect of naringenin on NLRP3 inflammasome is not prominent in flavonoids, suggesting that the dihydroflavone skeleton may have a unique advantage in anti‐pyroptosis. However, the sub‐compounds are mostly in the form of glycosides, and the oral bioavailability is extremely low, which is the most important bottleneck restricting its efficacy. Nano‐delivery system and prodrug design are the key directions to break through this obstacle [75].

### 3.4 Isoflavones

The defining feature of isoflavones is that the B‐ring attaches itself to the C‐ring at the third position instead of the usual second position which gives it a distinct spatial configuration and estrogenic activity.

#### 3.4.1 Genistein

Genistein, also known as genistein, is a naturally occurring isoflavone compound mainly found in legumes, especially in soybeans and their products. It is one of the main components of soybean isoflavones and is called “phytoestrogen” because of its similar structure to estrogen [76]. Estrogen receptors (ERα and ERβ) are expressed in articular chondrocytes, and estrogen signaling plays a protective role in cartilage homeostasis. Genistein, like other isoflavones, can bind to ERα and ERβ, but has a higher affinity for ERβ. After binding, the ER‐ligand complex is translocated to the nucleus and regulates the transcription of target genes containing estrogen response elements (ERE) [77, 78]. This leads to up‐regulation of cartilage matrix proteins (type II collagen and aggrecan), down‐regulation of pro‐inflammatory cytokines and MMP, and promotes chondrocyte survival by regulating apoptosis‐related genes [79]. In addition, ER signaling can activate the PI3K/Akt and MAPK cascades through the rapid non‐genomic effects of membrane‐associated ER. Mediating NF‐kβ inhibition prevents NF‐kβ nuclear translocation through protein–protein interaction, it promotes the expression of growth factors such as TGF‐β and IGF‐1, thereby supporting cartilage repair [80].

Postmenopausal estrogen deficiency is a recognized risk factor for accelerated progression of OA in women, so the ER regulation characteristics of isoflavones are particularly relevant. Several clinical studies have explored the application of soy isoflavone supplements in postmenopausal women with OA, and some studies have reported mild improvements in pain and joint function. However, the results are not consistent, and the optimal dose, preparation, and patient selection criteria are still unclear [81]. Isoflavones have a relatively weak estrogenic activity compared to endogenous estrogens, which means that they may provide benefits in an estrogen‐deficient state without producing hormone replacement therapy‐related adverse reactions [82, 83]. These multiple mechanisms make it have significant anti‐inflammatory and repair‐promoting functions, especially for postmenopausal OA.

#### 3.4.2 Daidzein

Daidzein is another major active ingredient of soybean isoflavones. It has anti‐inflammatory, antioxidant, and bone protective effects. Compared with genistein, its 5‐position has no hydroxyl group. It down‐regulated the expression of MMPs, IL‐6, and iNOS by blocking NF‐κB nuclear translocation and inhibiting MAPK phosphorylation. In the IL‐1β‐induced chondrocyte model, daidzein enhances cell defense by activating the Nrf2/ARE pathway and up‐regulating SIRT1 expression [84]. In vivo experiments have confirmed that daidzein can effectively alleviate the progress of rat OA model, and its mechanism includes bidirectional regulation of Wnt/β‐catenin pathway and improvement of OPG/RANKL balance [85].

#### 3.4.3 Other Isoflavone Compounds

**FIGURE 6:**
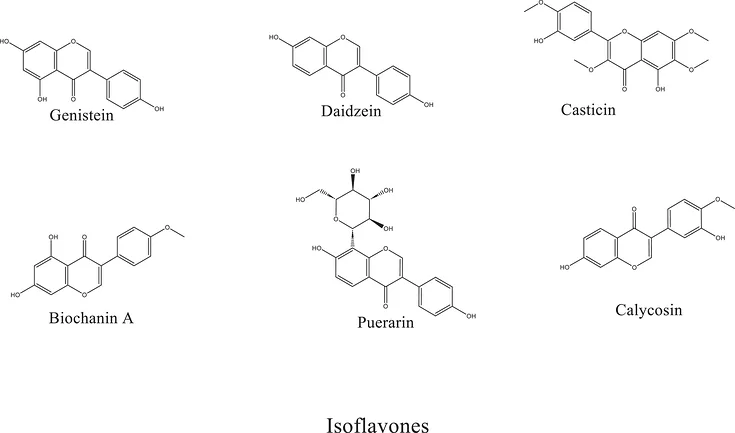
Chemical structures of representative isoflavones. (All chemical structures were drawn using ChemDraw (Version 22.0, CambridgeSoft).

Other flavonoid alkane compounds also show multi‐target effects in the prevention and treatment of OA. Genistein inhibited the production of NO, MMP‐2, and MMP‐9, and reduced ROS production by restoring Nrf‐2/HO‐1 expression to reduce joint inflammation [77]; casticin inhibits NF‐κB pathway, down‐regulates MMP‐13 and inflammatory mediators, and increases antioxidant activities such as GSH/GSG and SOD [86]; biochanin A is involved in the regulation of ferroptosis: the former inhibits chondrocyte ferroptosis by up‐regulating Nrf2 and GPX4 and down‐regulating MMP3; the latter reduced iron content and ROS accumulation by regulating FPN, TfR1, and system‐xc/GPX4 pathways. Puerarin can reduce inflammatory factors such as PGE2, IL‐6, and TNF‐α and increase GPX4 expression, reduce oxidative stress, and iron overload [87]; calycosin inhibits inflammatory response by regulating related inflammatory pathways [88]. The above mechanism of action has been verified in related cell models and various animal OA models. It is worth noting that puerarin (8‐C‐glucose‐7,4' ‐dihydroxyisoflavone) is a C‐glycoside. Unlike common O‐glycosides, C‐glycosidic bonds are more resistant to acid and intestinal enzyme hydrolysis, and therefore have unique pharmacokinetic characteristics (Figure 6).

Because of its unique 3‐phenylchromone structure, isoflavones are different from other flavonoid subclasses and have significant estrogen receptor regulatory activity, which provides a targeted advantage for the treatment of postmenopausal OA. The 5,7,4'‐trihydroxy structure of genistein makes its antioxidant and estrogenic activities stronger than that of daidzein, and the preclinical evidence is more sufficient. However, estrogen‐like effects also bring concerns about the safety of hormone‐sensitive tissues, which needs to be paid attention to in development and clinical application [89].

### 3.5 Flavanols

The characteristics of flavanols are that the C‐ring is a saturated bond and there are hydroxyl groups at the 3‐position, but there is no carbonyl group at the 4‐position, which mainly exists in the form of monomer or polymer (proanthocyanidin).

#### 3.5.1 Epigallocatechin Gallate (EGCG)

As the most active catechin in green tea, EGCG is famous for its strong antioxidant and anti‐inflammatory properties. Its structure is (2R, 3R) ‐5,7,3',4',5'‐pentahydroxyflavan‐3‐ol 3‐gallate. The gallic acyl group in its structure and the o‐trihydroxyl group in the B‐ring are the key to its strong biological activity. It plays a synergistic role through multiple targets: on the one hand, by inhibiting NF‐κB, JNK/MAPK, and other inflammatory pathways, it significantly down‐regulates the production of COX‐2, iNOS and a variety of pro‐inflammatory factors and catabolic enzymes [90, 91]. On the other hand, activation of Keap1/Nrf2/ARE and AMPK/mTOR pathways effectively enhances cellular antioxidant defenses and induces protective autophagy [92, 93]. It is worth noting that EGCG transcends the classical pathway and can regulate from the epigenetic level (such as down‐regulation of miR‐140‐3p to inhibit ADAMTS5) and metabolic level (inhibition of AGEs‐induced advanced glycation end products) [94, 95, 96], and inhibit abnormal Wnt/β‐catenin signaling to reduce osteophyte formation [90].

Although the strong biological activity of EGCG has been widely recognized, it has chemical instability and oxidizability. EGCG is prone to epimerization, oxidation, and degradation in aqueous solution, especially under neutral to alkaline pH conditions, thereby significantly shortening its half‐life and reducing bioavailability. In addition, several case reports have shown that high doses of green tea extract (usually EGCG daily dose of more than 800 mg) are associated with hepatotoxicity, and the mechanism may involve mitochondrial toxicity, oxidative stress, and idiosyncratic drug reactions [97]. This highlights the need for rigorous dose exploration studies and safety monitoring in any clinical development plan. Therefore, although EGCG has shown significant potential in preclinical studies, its clinical transformation must be properly addressed through appropriate formulation strategies and careful dose optimization to address these stability and safety challenges [98].

#### 3.5.2 Procyanidin B2

Proanthocyanidin B2 is a dimer composed of two (epi) catechin units connected by C4─C8 bonds. It is a proanthocyanidin dimer widely found in grape seeds, blueberries and other plants, which has excellent antioxidant and anti‐inflammatory activities [99]. This leads to the down‐regulation of the expression of MMPs, ADAMTS, and inflammatory factors through inhibition of NF‐κB and JNK/ERK pathways. In the IL‐1β‐induced chondrocyte degeneration model, procyanidin B2 promotes protective autophagy by activating the PI3K/Akt/mTOR pathway and enhances the activity of the Nrf2/ARE pathway. In vivo studies have confirmed that it can significantly alleviate the progression of OA in rats by regulating TGF‐β/Smad signaling and improving the balance of OPG/RANKL system [100].

#### 3.5.3 Other Flavanol Compounds

**FIGURE 7:**
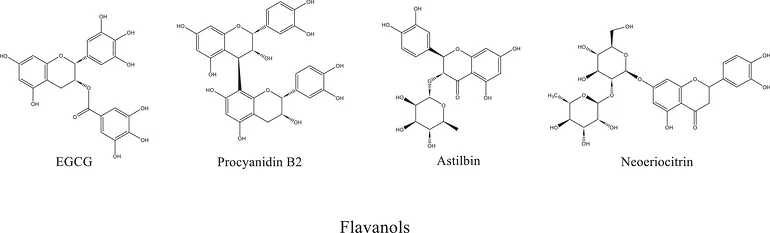
Chemical structures of representative flavanols. (All chemical structures were drawn using ChemDraw (Version 22.0, CambridgeSoft).

The difference in the activity of other flavanol compounds is closely related to the hydroxylation mode and degree of polymerization of the monomer. Orientin glycosides can reduce chondrocyte senescence by inhibiting the PI3K/AKT pathway [101]. Isoorientinapioside can significantly reduce the bacterial load and swelling of joints, mainly by regulating inflammatory pathways; astilbin may inhibit the inflammatory response through the TLR/MyD88/NLRP3 pathway; in addition to anti‐inflammatory and anti‐oxidation, neoeriocitrin also has a unique role in promoting osteogenic differentiation and enhancing bone regeneration (Figure 7).

In general, flavanol monomers have one of the strongest antioxidant and anti‐inflammatory activities, and their galloyl and o‐triphenol hydroxyl groups are the key to activity. However, these structural characteristics also lead to its extremely unstable, easy oxidative polymerization, and little intestinal absorption after oral administration, most of which is degraded by the flora in the colon [102]. Although the proanthocyanidin polymer has significant activity in vitro, it is hardly absorbed as a macromolecule, and its effect may be more dependent on intestinal metabolites [98]. Therefore, flavanols are facing severe challenges as oral OA therapeutic drugs, and are more suitable as functional food ingredients or developed as intra‐articular injection preparations.

### 3.6 Anthocyanins

Anthocyanins are compounds with yellow salt cation (2‐phenylbenzopyran) as the parent nucleus. Their color and reactivity change with pH value and have strong antioxidant capacity.

#### 3.6.1 Cyanidin

Cyanidin is the main anthocyanin in dark berries such as blackberries and blueberries. It usually exists in the form of glycosides, and its structure is 3,5,7,3',4'‐pentahydroxy yellow salt. The catechol hydroxyl group of the B‐ring is the basis of its strong antioxidant activity, showing significant anti‐inflammatory and cytoprotective effects in the treatment of OA. Its core mechanism is to effectively down‐regulate the expression of key inflammatory mediators such as MMPs, IL‐6, TNF‐α, and iNOS by inhibiting NF‐κB and p38/ERK signaling pathways, thereby reducing joint inflammation and cartilage destruction [103, 104, 105]. At the same time, it synergistically enhances the antioxidant defense ability of cells and promotes protective autophagy by activating the Nrf2/HO‐1 and AMPK/SIRT1 signaling axes. It is particularly noteworthy that cyanidin can effectively protect chondrocytes through the SIRT1/autophagy pathway in metabolic stress environments such as high glucose, which makes it have potential specific application value for OA related to metabolic abnormalities such as diabetes [106]. Animal studies have further confirmed that it can effectively alleviate the progression of OA in rats induced by various factors by inhibiting abnormal Wnt/β‐catenin pathway activation and regulating PI3K/Akt/mTOR pathway.

#### 3.6.2 Delphinidin

Delphinidin is an anthocyanin that gives plants a blue hue and also exists in the form of glycosides. Its B‐ring has 3', 4', 5'‐trihydroxyl. It can selectively inhibit JNK/p38 MAPK and NF‐κB pathways and down‐regulate effector molecules such as MMP‐3, MMP‐13, IL‐1β, TNF‐α, and PGE2. In the inflammatory cell model, delphinidin exerts a protective effect by activating the Nrf2/ARE pathway and inhibiting the assembly of NLRP3 inflammasome [73]. In vivo studies have shown that delphinidin can regulate the balance of PI3K/Akt and Wnt/β‐catenin pathways to alleviate cartilage damage and osteophyte formation in rat OA models.

#### 3.6.3 Pelargonidin

Pelargonium is bright orange–red or brick red, and the red tone of its glycosides is usually more orange than that of cyanidin. It is mainly derived from strawberries, pomegranates, mature red mulberry, and so forth. Its B‐ring has only one 4'‐hydroxyl, so its antioxidant capacity is weaker than cyanidin and delphinidin. Current studies based on cell and animal models have shown that pelargonidin has a potential protective effect on OA [107]. The core mechanism is to reduce joint inflammation by inhibiting key inflammatory pathways such as NF‐κB; it can also directly activate the Nrf2 defense system and alleviate the damage of oxidative stress to cartilage. At the level of cartilage protection, it can effectively down‐regulate the expression of matrix degradation enzymes and promote the synthesis of collagen II and proteoglycans to maintain the integrity and function of cartilage matrix.

#### 3.6.4 Malvidin

Malvidin is dark purple to blue purple, which is the most important anthocyanin in red wine and also a dimethylated derivative of delphinidin. Existing experiments have shown that malvidin has certain potential in the intervention of OA. It not only exerts anti‐inflammatory effects by potently inhibiting NF‐κB and MAPK pathways, but also directly scavenges free radicals and activates endogenous antioxidant responses. In particular, malvidin might be able to inhibit inflammatory processes and relieve the cell senescence by activating the deacetylase SIRT1 pathway [103], and has the effect of inhibiting osteoclast formation, which is helpful to maintain the stability of subchondral bone.

**FIGURE 8:**
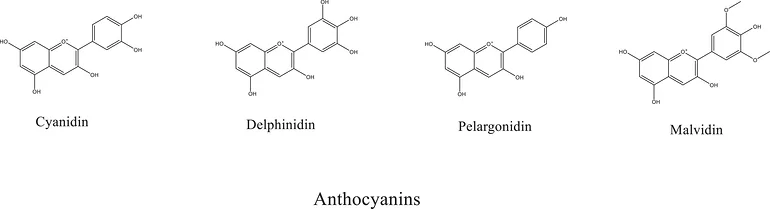
Chemical structures of representative anthocyanins. (All chemical structures were drawn using ChemDraw (Version 22.0, CambridgeSoft).

In addition, recent studies have revealed a new dimension of anthocyanin action‐“intestinal‐articular axis.” Anthocyanins can be metabolized by intestinal flora into small molecular phenolic acids with higher biological activity in vivo. These metabolites can indirectly affect the OA process by regulating systemic inflammation and oxidative stress levels [108]. For example, bacterial metabolites of cyanidin‐3‐glucoside have been shown to inhibit systemic inflammatory factors and thus have a positive impact on joint health [109] (Figure 8).

The antioxidant activity of anthocyanins is positively correlated with the degree of B‐ring hydroxylation (delphinidin >cyanidin >pelargonidin), but its cationic structure is extremely unstable at physiological pH, and oral bioavailability is usually less than 1%. Its clinical effect may be more dependent on intestinal microbial metabolites than prototype molecules [110]. Therefore, anthocyanins are more suitable as functional food ingredients for OA‐assisted interventions, rather than as traditional oral drugs.

### 3.7 Chalcones

The chalcones are the precursors to flavonoid biosynthesis. Its structural characteristic is that the C‐ring is not a closed one, and it is an open α,β‐unsaturated ketone system. The open‐chain structure provides it with some special flexibility and electrophilicity.

#### 3.7.1 Isoliquiritigenin

Isoliquiritigenin is a natural chalcone compound extracted from licorice, and it is also one of the most studied chalcones at present. Its unique α,β‐unsaturated ketone structure can be used as a Michael addition receptor to covalently bind to the cysteine sulfhydryl group in some signal proteins, thereby regulating its activity. In the treatment of OA [111]: Isoliquiritigenin can not only significantly reduce the expression of IL‐1β, TNF‐α, PGE2, and MMPs by inhibiting key inflammatory signaling pathways such as NF‐κB and MAPKs, thereby reducing cartilage degradation and joint inflammation, but also has strong free radical scavenging ability, which can reduce the damage of oxidative stress to chondrocytes and maintain the normal function of chondrocytes. In addition, it can effectively inhibit the apoptosis of chondrocytes induced by inflammatory factors, maintain the number and vitality of chondrocytes, and delay the progression of OA [112]. At present, a large number of in vitro cell and animal OA model studies have confirmed that isoliquiritigenin can effectively reduce joint inflammation, slow down cartilage degeneration and synovial hyperplasia, and is a popular natural candidate molecule for the development of OA treatment drugs.

#### 3.7.2 Phloretin

Phloretin is one of the most classic chalcones, mainly derived from apple peel, apple leaves, and some Rosaceae plants. It has a long history of research and has a wide range of biological activities. Phloretin is a natural compound with high antioxidant properties, which can serve as a scavenger and alleviate oxidative stress [113]. Simultaneously, it can be quite effective in suppressing the expression of inflammatory mediators and matrix metalloproteinases and decrease both joint inflammation and cartilage degeneration. In OA research, phloretin has been shown to protect chondrocytes, reduce inflammation‐mediated extracellular matrix degradation, maintain cartilage structural, and functional integrity. As a classical chalcone, phloretin provides an important reference for understanding the SAR and mechanism of chalcone compounds, and also provides a candidate molecule for the development of new OA treatment drugs.

#### 3.7.3 Sappanone A

Sappanone A is a bioactive chalcone extracted in *Caesalpinia sappan*, which has recently drawn attention because of its special mechanism in relation to chondrocyte ferroptosis in OA. The main protective property of sappanone A is to be able to regularly stimulate intracellular antioxidant defense and ferroptosis inhibition networks [114]. It is able to increase the expression of transcription factor Nrf2 substantially, which leads to the increased transcription of its downstream target genes, increased anti‐oxidative stress capacity of the cells, and removal of excess reactive oxygen species. Sappanone A may also increase the expression of the deacetylase SIRT1, preserve mitochondrial integrity, prevent apoptosis, and create a synergistic relationship with the Nrf2 pathway to help improve cell homoeostasis. Furthermore, sappanone A may also increase the synthesis of type II collagen. The model of ferroptosis of chondrocytes caused by IL‐1 b has been used and it has been shown that sappanone A treatment is capable of reversing the down‐regulation of such important proteins and cell viability. Its usage has also demonstrated the possibility to significantly decrease cartilage damage and synovial inflammation in animal models of OA, and thus has a good potential in protecting cartilage by acting on multiple targets in the ferroptosis pathway.

#### 3.7.4 Cardamonin

**FIGURE 9:**
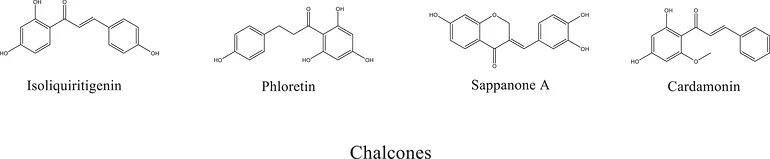
Chemical structures of representative chalcones. (All chemical structures were drawn using ChemDraw (Version 22.0, CambridgeSoft).

Cardamonin is a natural chalcone derived from cardamom plants. The main mechanism of its treatment of OA is the dual role of regulating p53 signaling pathway and inhibiting chondrocyte ferroptosis [115]. Studies have shown that cardamonin can regulate the activity of p53 signaling pathway, effectively reverse the inhibition of SLC7A11 and GPX4 gene expression by IL‐1β, and restore the antioxidant and anti‐ferroptosis ability of cells. Cardamonin can reduce the inhibition of IL‐1β on COL2A1 gene expression and promote the synthesis of cartilage characteristic matrix through its synergistic effect of anti‐inflammatory and anti‐ferroptosis. In the two classic rat OA models of ACLT and DMM, cardamonin intervention can observe positive changes such as more complete cartilage surface, reduced proteoglycan loss, and improved subchondral bone sclerosis [116]. These together indicate that cardamonin inhibits ferroptosis through the p53‐SLC7A11/GPX4 axis and protects the extracellular matrix, thereby delaying the cartilage degeneration process of OA (Figure 9 and Table 1).

**TABLE 1:** Experimental evidence for the potential of flavonoids in the treatment of osteoarthritis.

Category	Compound	Main natural sources	Experimental model and dose	Key molecular targets	Research limitations	Document
Flavonoids	Luteolin	Celery, broccoli, honeysuckle	IL‐1β‐induced rat chondrocytes (10 µM), rat OA model	↓NF‐kβ,↓MAPK,↓MMP‐13,↑type II collagen,↑AMPK‐Nrf2	Most of them are preclinical studies, pharmacokinetic data are limited	[15, 16, 17, 18, 19, 20]
	Apigenin	Parsley, Citrus fruits	Human chondrocytes, mouse OA model	↓NF‐kβ,↓MAPK,↑M2macrophages,↑type II collagen,↓MMP‐13,↓HIF‐2α	The in vivo efficacy needs further verification.	[21, 22, 23, 24, 25]
	Baicalein	*Scutellaria baicalensis* (root)	RAW264.7 macrophages, osteoclast culture	↓NF‐kβ,↓p38/JNK,↓PGE2,↓NO,↓osteoclast differentiation	Limited animal model data	[26, 27, 28, 29]
	Kaempferol 3‐O‐glucoside	Membranous milkvetch root	IL‐1β‐induced rat chondrocytes (25–100 µM), ACLT rat model (40 mg/kg/day, gavage)	↓NF‐kβ,↓MAPK,↓Inflammatory mediators	Limited to a single animal model	[30]
	Oroxin B	Wooden butterfly (plant)	IL‐1β‐induced mouse chondrocytes (5–20 µM), DMM mouse model (10‐20 mg/kg, intraperitoneal injection)	↓MMP‐3,↓MMP‐13,↓ADAMTS5,↓COX‐2	The dose‐effect relationship has not been fully explored.	[31, 32]
	Nobiletin	Peel of citrus fruits	IL‐1β‐induced rat chondrocytes (25–100 µM), aCLT rat model (50 mg/kg/day, gavage)	↓NF‐kβ,↓PI3K/Akt,↓inflammation	Lack of long‐term efficacy data	[33]
	Baicalin	Baikal skullcap root	LPS‐induced mouse macrophages (10–50 µM), Rat OA model (100 mg/kg/day, intragastric administration)	↓TLR4/NF‐κB,↑M2 macrophages	Bioavailability issues	[29]
	Tilianin	Thistle (plant)	IL‐1β‐induced rat chondrocytes (10–40 µM), rat OA model (10‐40 mg/kg/day, gavage)	↓TLR4/NF‐Kβ,↓oxidative stress,↓apoptosis	The depth of the mechanism is limited	[33]
Flavonols	Quercetin	Onions, apples, tea	Human chondrocytes (10–100 µM), rat OA model (50 mg/kg/day, oral), clinical trials	↓NF‐kβ,↑Nrf2/HO‐1,↑AMPK/Nrf2/GPX4,↓MMPs,↑type II collagen	There are differences in clinical results; low oral bioavailability	[34, 35, 36, 37, 38, 39]
	Kaempferol	Spinach, green tea	IL‐1β‐induced Rat chondrocytes (5–40 µM), mouse/canine OA model (20–100 mg/kg/day)	↓NF‐kβ,↓MAPK,↓PI3K/Akt/mTOR,↑Nrf2/HO‐1, regulating XIST/miR‐130a/STAT3 axis	Study heterogeneity in meta‐analysis	[40, 41, 42, 43, 44]
	Wogonin	*Scutellaria baicalensis* (root)	IL‐1β‐induced SW1353 cells(10–50 µM)	↓AP‐1,↓JAK2/STAT1/2,↓MMP‐13	Only cell line data	[47, 48]
	Hesperetin	Peel of citrus fruits	DMM mouse model (50 mg/kg/day, gavage)	↑Nrf2/HO‐1,↓NF‐kβ	Single animal model	[49]
	lcariin	Epimedium (plant)	TNFα‐induced ADTC5 mouse chondrocytes and male SD rat articular chondrocytes (10–50 µM)	↓NF‐kβ/HIF‐2α,↓MMP‐9,↓ADAMTS‐5	Lack of in vivo efficacy data	[50]
	Myrtillin	Myrtle (plant)	IL‐1β‐induced mouse chondrocytes (10–50 µM)	↓PI3K/Akt/NF‐κB,↓MAPK	Only in vitro data	[40, 41]
	Diosmetin	Citrus, celery and other plants.	LPS‐induced macrophages (10–40 µM), rat OA model (20–80 mg/kg/day, gavage)	↓PI3K/AKT,↑M2 macrophages,↓Inflammation	Dose optimization needs	[51]
	Hyperoside	Hypericum perforatum (Hypericum)	IL‐1β‐induced rat chondrocytes (10–40 µM), rat OA model (10‐40 mg/kg/day, intragastric administration)	↓PI3K/AKT/NF‐kβ,↓Inflammation, anti‐oxidation, antiapoptosis	Research on the mechanism is limited	[40, 41]
	Fisetin	Lacqueraceous plants (e.g., mango)	Human chondrocytes (5–20 µM), mouse OA model (10–20 mg/kg, intraperitoneal injection)	↑SIRT6/Nrf2/HO‐1,↓Chondrocyte senescence	Dose standardization needs	[40, 41]
	Isoquercetin	Sea buckthorn, ginkgo, etc.	Rat chondrocytes induced by hydrogen peroxide (10–50 µM), rat OA model (10–40 mg /kg/day, intragastric administration)	↓NOX4/Nrf2,↑redox balance,↓aging	Single oxidative stress model	[52]
	Astragalin	Milk vetch (astragalus plants)	IL‐1β‐induced rat chondrocytes (5–20 µM), rat OA model (20–80 mg/kg/day, gavage)	↓JNK/p38 MAPK,↓MMP3,↓Inflammatory factors	Limited in vivo pharmacokinetic data	[40, 41]
	Rutin	Buckwheat, Sophora, Ruthenium	IL‐1β‐induced rat chondrocytes (10–100 µM), rat OA model (50‐200 mg/kg/day, gavage)	↓Inflammatory pathway	Low bioavailability; high dose is required	[10, 11]
Flavanones	Hesperidin	Peel of citrus fruits	IL‐1β‐induced chondrocyte model (10–100 µM)	↓NF‐kβ,↓MAPK,↓MMPs,↓PGE2/NO,↑PI3K/Akt	Lack of in vivo efficacy and pharmacokinetic data	[49, 53, 54]
	Naringenin	Hesperidium	Rat chondrocytes (20–100 µM), ACLT/MIA rat model (50‐100 mg/kg/day, gavage)	↓NF‐kβ,↓MAPK,↑SIRT1 mediated autophagy,↓NLRP3,↑TGF‐β/Smad3	Pharmacokinetic challenges; nano‐preparations showed improved efficacy	[55, 56]
	Eriocitrin	Lemon, Lime	Rat chondrocytes (10–50 µM), rat OA model (50 mg/kg/day, intragastric administration)	↓NF‐kβ,↓MAPK,↑Nrf2/HO‐1,↓MMPs, ↑type II collagen/SOX9	Metabolism into eriodictyol in vivo	[59]
Isoflavones	Genistein	Soybean and its products	Chondrocytes (10–100 µM), ovariectomized rat OA model (50 mg/kg/day, gavage).	↓NF‐kβ,↓MAPK,↑PI3K/Akt/mTOR,↑autophagy,↑Wnt/β‐catenin,↑OPG/RANKL	Estrogen‐like effects need to be carefully evaluated	[61, 62, 63, 64, 65]
	Daidzein	Soybean and its products	IL‐1β‐induced rat chondrocytes (10–80 µM), rat OA model (50 mg/kg/day, intragastric administration)	↓NF‐kβ,↓MAPK,↑Nrf2/ARE,↑SIRT1,↓MMPs,↓IL‐6/iNOS	The curative effect in vivo is not as significant as genistein.	[66, 67]
	Casticin	*Viticis* plants	IL‐1β‐treated mouse ADTC5 cells (5–20 µM)	↓NF‐kβ,↓MMP‐13,↑GSH/GSSG,↑SOD	Only cell line data	[68]
	Biochanin A	Chick‐pea	DMM mouse model (20‐50 mg /kg/day, intraperitoneal injection)	↑Nrf2/GPX4,↓MMP3,↓FPN,↓TfR1,↓ROS	Model specificity (iron overload)	[40, 41]
	Puerarin	Lobed kudzuvine root	ACLT rat OA model (100 mg/kg/day, gavage)	↓PGE2,↓IL‐6,↓TNF‐α,↑GPX4,↓Oxidative stress	Need high dose, the pharmacokinetics of C‐glycosides are different.	[69, 70]
	Calycosin	*Astragalus membranaceus* and other legumes	IL‐1β‐induced rat chondrocytes (10–40 µM), rat OA model (10–40 mg/kg/day, gavage)	↓Inflammatory pathway	The depth of the mechanism is limited	[40, 41]
Flavanols	EGCG	Green tea	Chondrocytes (10–100 µM), rat/mouse OA model (50‐100 mg/kg/day, gavage/intraperitoneal injection), clinical trials	↓NF‐kβ,↓JNK/MAPK,↑Keap1/Nrf2/ARE,↑AMPK/mTOR,↓miR‐140‐3p/ADAMTS5,↓AGEs,↓Wnt/β‐catenin	Limited clinical trial data, there are differences in efficacy	[71, 72, 73, 74, 75, 76, 77, 78]
	Procyanidin B2	Grape seeds, blueberries	IL‐1β‐induced rat chondrocytes (10–80 µM), rat OA model (50 mg/kg/day, intragastric administration)	↓NF‐kβ,↓JNK/ERK,↑PI3K/Akt/mTOR,↑Nrf2/ARE,↓MMPs/ADAMTS,↑TGF‐β/Smad,↑OPG/RANKL	dimer specific activity, higher polymer not studied	[79, 80]
	Astilbin	*Astilbe* (plant)	LPS‐induced macrophage inflammation model, animal OA model Macrophages (10–50 µM), rat OA model (20–80 mg/kg/day, gavage)	↓TLR,↓TLR/MyD88/NLRP3,↓inflammation	Limited in vivo data	[40, 41]
	Neoeriocitrin	North American holy grass (plant)	BMSCs (10–50 µM), rat bone defect model (10–40 mg/kg/day)	↑osteogenic differentiation,↑bone regeneration	The specific effect of OA needs to be verified.	[40, 41]
Anthocyanins	cyanidin	Blackberry, blueberry	Rat chondrocytes induced by high glucose (10–80 µM), rat OA model (50‐100 mg/kg/day, intragastric administration)	↓NF‐kβ,↓p38/ERK,↑Nrf2/HO‐1,↑AMPK/SIRT1,↓MMPs/IL‐6/TNF‐α/iNOS,↓Wnt/β‐catenin	It has potential value for metabolic OA (diabetes).	[82, 83, 84, 85, 86]
	Delphinidin	Blue flowers and berries	Inflammatory cell model (10–50 µM), Rat OA model (50 mg/kg/day, gavage)	↓JNK/p38 MAPK,↓NF‐kβ,↑Nrf2/ARE,↓NLRP3,↓MMP‐3/MMP‐13/IL‐1β/TNF‐α/PGE2	pH‐sensitive stability	[59]
	Pelargonidin	Strawberry, red onion, pomegranate	Inflammatory cell model (10–50 µM), Rat OA model (50 mg/kg/day, gavage)	NF‐kβ,↑Nrf2,↓matrix‐degrading enzyme,↑type II collagen	Antioxidant activity is weak	[87]
	Malvidin	Red wine (especially aged red wine), purple sweet potato, purple corn	Inflammatory cell model (10–80 µM), osteoclast culture	↓NF‐kβ,↓MAPK,↑SIRT1,↓inflammation,↓osteoclast formation	Lack of in vivo OA model data	[82, 83, 88]
Chalcones	Isoliquiritigenin	Licorice root	Chondrocytes (5–40 µM), rat OA model (20‐50 mg kg/day, gavage)	↓NF‐κB,↓MAPK,↓IL‐1β/TNF‐α/PGE2/MMPs,↓oxidative stress,↓apoptosis	Multi‐target effect; need for target selectivity research	[90, 91]
	Phloretin	Apple peel, apple leaves	Chondrocytes (10–80 µM, rat OA model (50 mg/kg/day, intragastric administration)	↓Inflammatory mediators,↓MMPs,↓oxidative stress	Research on the mechanism is limited	[59]
	Sappanone A	Sumu (plant)	IL‐1β‐induced rat chondrocytes (5–20 µM), rat OA model (10–40 mg/kg/day, intragastric administration)	↑Nrf2,↑SIRT1,↓ferroptosis,↑type II collagen	Emerging compounds; need to verify	[87]
	Cardamonin	Cardamom	ACLT/DMM rat model (25‐50 mg/kg/day, gavage).	↑p53‐SLC7A11/GPX4 axis,↓ferroptosis,↑COL2A1,↓ECM degradation	Lack of in vitro mechanism data	[90, 91]

In summary, the α,β‐unsaturated carbonyl system in chalcone can be used as a Michael acceptor to covalently modify the nucleophilic cysteine residues in the target protein. This chemical feature is particularly noteworthy for the activation of the Nrf2/ARE antioxidant pathway. Keap1 is a negative regulator of Nrf2.Chalcone can covalently modify specific cysteine residues (Cys151, Cys273, and Cys288) in Kelch‐like ECH‐related protein 1 (Keap1). This modification induces the conformational change of Keap1, disrupts the Keap1‐Nrf2 interaction, translocates Nrf2 to the nucleus, and initiates the transcription of antioxidant response element (ARE)‐regulated genes [117]. Molecular docking studies suggest that isoliquiritigenin and other chalcones can bind well to the Kelch domain of Keap1 through hydrophobic interactions and hydrogen bonds, and their α,β‐unsaturated carbonyl groups can be positioned to promote the Michael addition of key cysteine thiols. Although this electrophilic property is interesting in mechanism, it also raises potential safety concerns about off‐target reactivity and protein alkylation, which should be carefully evaluated in toxicity studies.

## 4 Structure–Activity Relationship, Action Basis and Clinical Transformation of Flavonoids

**FIGURE 10:**
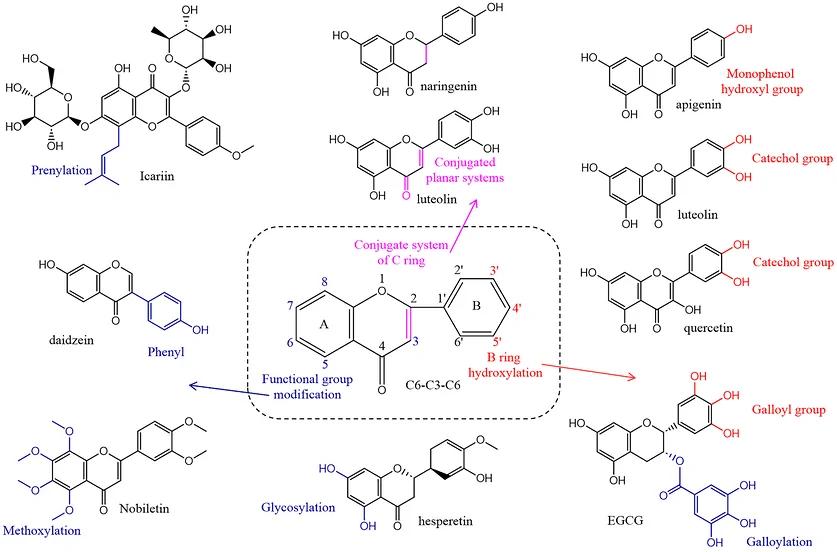
Synopsis of structure–activity relationship (SAR) of anti‐osteoarthritis active flavonoids. (All chemical structures were drawn using ChemDraw (Version 22.0, CambridgeSoft).

The potential of flavonoids to prevent and treat OA stems from the close relationship between the diversity of their chemical structures and their biological activities. Although the above flavonoids share the basic skeleton of C6─C3─C6, the subtle differences in parent nucleus type, functional group modification and stereo configuration profoundly affect their antioxidant capacity, anti‐inflammatory activity, target selectivity, and pharmacokinetic properties, thus determining the diversity and intensity of their anti‐OA effects (Figure 10).

### 4.1 Effects of Structural Characteristics on Antioxidant Activity

Antioxidant activity is an important basis for flavonoids to exert cartilage protection, and its core lies in scavenging free radicals and chelating metal ions. A large number of studies have shown that the structure of catechol group (3',4'‐di‐OH) or galloyl group (3',4',5'‐tri‐OH) on the B‐ring is a decisive factor in determining its antioxidant capacity [118]. The catechol hydroxyl group can stabilize the phenoxy radicals generated after oxidation by forming intramolecular hydrogen bonds, thereby efficiently terminating the free radical chain reaction. For example, luteolin, quercetin, cyanidin, and eriodictyol all exhibit excellent free radical scavenging ability due to the presence of this structure. The o‐triphenol hydroxyl group provides additional hydroxyl groups, which further enhances the electron‐donating ability and metal ion chelating ability, making the antioxidant activity stronger. EGCG is one of the flavonoids with the strongest antioxidant activity due to its o‐triphenolic hydroxyl group and gallic acyl group on the B‐ring [119]. If there is only one phenolic hydroxyl group (apigenin, naringenin) or no hydroxyl group on the B‐ring, its antioxidant capacity will be significantly reduced. This explains why the activity of apigenin on some anti‐inflammatory pathways is comparable to that of luteolin, but its ability to directly scavenge ROS is far inferior to the latter.

### 4.2 Effects of Structural Features on Molecular Planarity and Target Binding

The planarity of the parent nucleus of flavonoids directly affects its binding affinity to the hydrophobic pocket of target proteins (kinases and transcription factors), thereby affecting anti‐inflammatory activity. In flavones and flavonols, the C2═C3 double bond forms a conjugated system with the 4‐carbonyl group so that the entire C‐ring and the connected B‐ring are basically in the same plane (rigid plane structure). This planar structure facilitates the insertion of compounds into the catalytic pockets or receptor binding domains of certain enzymes, such as the subunits of MAPK and NF‐κB, thereby more effectively inhibiting their activity [120]. For example, the broad‐spectrum anti‐inflammatory activity of luteolin and quercetin is partly attributed to this. In contrast, flavanones and flavanonols have a nonplanar, twisted butterfly‐shaped conformation due to the C2‐C3 single bond and the folding of the C‐ring. Although this conformational change may weaken some broad‐spectrum anti‐inflammatory activities, it may enhance the selectivity of specific targets. For example, the inhibitory effect of naringenin on NLRP3 inflammasome is considered to be related to its nonplanar conformation that better adapts to the binding site of the target [121].

### 4.3 Effects of Structural Characteristics on Pharmacokinetics

The absorption, distribution, metabolism, and excretion (ADME) characteristics of flavonoids, especially oral bioavailability, are the main bottlenecks in their clinical transformation. The introduction of saccharide groups (hesperidin and rutin) can significantly increase the water solubility of the compound, which is conducive to the development of the preparation. However, the presence of glycosyl groups increases molecular weight, reduces membrane permeability, and makes it a substrate for efflux pumps (P‐gp), resulting in extremely low oral bioavailability. These compounds are usually regarded as “prodrugs” and need to be hydrolyzed by intestinal flora or enzymes in the body to release aglycones (hesperetin and quercetin) before they can exert their main efficacy. Methylation of hydroxyl groups (nobiletin) can significantly increase the liposolubility of molecules and improve their ability to penetrate cell membranes. At the same time, methoxy groups can protect adjacent hydroxyl groups from rapid clearance of phase II metabolism (glucuronidation and sulfation), thereby improving oral bioavailability and metabolic stability [122]. This explains why polymethoxyflavonoids often exhibit excellent in vivo activity. Isopentenyl (8‐isopentenyl in icariin) is a potent lipophilic modification that can greatly increase the fat solubility of compounds and enhance their affinity with cell membranes or certain target proteins (enzymes involved in bone metabolism). Such modifications give the compounds unique potential to regulate bone metabolism. In EGCG, the introduction of galloyl groups not only provides additional phenolic hydroxyl groups to enhance antioxidant activity, but also affects its binding mode and metabolic pathway with proteins [119] (Table 2).

**TABLE 2:** Summarization of structure–activity relationship of anti‐osteoarthritis activity of flavonoids.

Structural characteristic	Location/Type	Main effects on anti‐OA activity	Mechanism/Pharmacokinetics (PK) significance	Representative compounds (activity difference)
B‐ring hydroxylation	Bisphenol hydroxyl (3',4'‐di‐OH)	The key to determining antioxidant activity	Efficient scavenging free radicals, chelating metal ions Enhance anti‐inflammatory effect	Luteolin, quercetin>apigenin, kaempferol
	O‐Trihydroxyl (3',4',5'‐tri‐OH)	The strongest antioxidant activity	Stronger free radical scavenging and metal chelating ability	EGCG
	Monophenolic hydroxyl (4'‐OH)	Antioxidant activity is weak	Mainly rely on anti‐inflammatory pathway to play a protective role	Apigenin, naringenin
C‐ring structure	C2═C3 double bond +4‐carbonyl (flavonoid/flavonol)	Enhance molecular rigidity and target affinity	A planar conjugated system is formed to facilitate the embedding of kinase/transcription factor hydrophobic pockets and enhance broad‐spectrum anti‐inflammatory activity.	Luteolin, quercetin
	C2─C3 single bond (dihydroflavone/dihydroflavonol)	Affect target selectivity	Twisting molecules may reduce broad‐spectrum activity but increase selectivity to specific targets	Naringenin, hesperetin
B‐ring connection position	3‐Phenyl (isoflavone)	Endow estrogen‐like activity	It can regulate cartilage metabolism by binding estrogen receptor (ER), which has special value for postmenopausal OA.	Daidzein, genistein
Functional group modification	Glycosylation (O‐glycosides)	Increased water solubility but decreased oral bioavailability	It is often used as a prodrug, and its efficacy depends on the hydrolysis of intestinal flora to release aglycones. Increase polarity and reduce membrane permeability	Hesperidin, rutin
	Methoxylation (Polymethoxy)	Improve oral bioavailability and metabolic stability	Increase fat solubility, improve membrane permeability Protect phenolic hydroxyl groups, reduce phase II metabolism and prolong half‐life	Naringin
	Isopentenylation (8‐prenyl)	Enhance fat solubility and specific target affinity	Increasing membrane affinity may enhance the regulation of bone metabolism‐related targets.	Icariin
	Gallic acylation (3‐galloyl)	Enhance antioxidant and protein binding capacity	Provide additional phenolic hydroxyl groups to increase the binding sites and modes with the target protein	EGCG

### 4.4 Clinical Transformation: Evidence, Challenges, and Future Directions

In terms of clinical transformation, a number of studies have preliminarily verified the safety and symptom improvement effects of some flavonoids. For example, a systematic review of quercetin[10]summarized its clear cartilage protection in preclinical models. The Cochrane systematic review of pine bark extract [123] and the clinical review of ginkgo biloba extract EGb 761 [124] have pointed out its positive effects in improving pain and function in patients with OA. However, it must be recognized that the vast majority of the evidence mentioned in this article comes from in vitro cell experiments and small animal models. Most of the existing clinical studies still focus on short‐term symptom improvement, lacking imaging evidence for long‐term repair of cartilage structure and safety data for large‐scale population applications [125, 126]. Although these models are indispensable for mechanism research, they cannot fully replicate the complexity of human OA pathophysiology, disease heterogeneity, and the placebo effect observed in the clinical environment. Despite encouraging progress in basic research, flavonoids still face multiple bottlenecks from the laboratory to the clinic (poor oral bioavailability, uncertain dose‐response relationship, hepatotoxicity risk at pharmacological doses, etc.).

In order to accelerate this transformation process, future research should focus on the following directions: Drug delivery systems based on targeted nanomedicine. The clinical application of flavonoids has long been limited by its inherent defects, such as low oral bioavailability, metabolic instability, and insufficient targeting. Nano‐delivery system provides a key technical path to solve these bottlenecks. In recent years, a number of innovative strategies with transformation prospects have emerged in this field. Li et al. self‐assembled proanthocyanidins (PAs) with strong antioxidant and anti‐inflammatory activities and Pluronic F127 (PF127) into injectable nanospheres through hydrogen bonding interactions. The nanospheres significantly increased cell viability in mouse L929 fibroblasts and ADTC5 chondrocytes, and enhanced the protein expression of COL1A1 and COL3A1 in fibroblasts and glycosaminoglycans and COL2A1 in chondrocytes. In the rat OA enzymatic hydrolysis model, PAs/PF127 nanospheres significantly reduced joint space swelling and accelerated the formation of subchondral bone and cartilage surface [127]. Gan et al. used two‐dimensional MXene nanosheets as a carrier to deliver quercetin. In IL‐1β‐induced primary mouse articular chondrocytes, MXene loading significantly enhanced the protective effect of quercetin, including improving cell viability and proliferation, reducing apoptosis, reducing oxidative stress, and inhibiting ferroptosis. In the OA mouse model, MXene‐quercetin treatment more effectively preserved cartilage integrity and inhibited ferroptosis than free quercetin [128]. In the future, the active targeting strategy of ligand modification and the development of synergistic nano‐delivery systems of flavonoids and other active ingredients or local long‐acting drug delivery preparations such as injectable hydrogels should be further explored to achieve accurate and efficient OA treatment [129].

The importance of research on metabolites derived from intestinal microflora. Recent studies have shown that gut microbiota and its metabolites are closely related to the occurrence, progression, and pathological process of OA. They may directly affect cartilage, or play an indirect role by affecting the intestinal, immune, and endocrine systems [129]. The in vivo activity of flavonoids not only depends on its parent structure, but also is closely related to its products after metabolic transformation by intestinal microorganisms. Glycosidic flavonoids need to be deglycosylated by intestinal flora to release active aglycones. In the OA rat model, quercetin treatment caused significant changes in the intestinal flora, including an increase in the proportion of *Clostridia*, a decrease in the proportion of *Bacilli*, and an increase in the level of *Lactobacillus*. It is worth noting that *Lactobacillus* has been reported to alleviate OA‐related pain and delay disease progression by inhibiting the production of pro‐inflammatory cytokines and reducing cartilage damage [130]. The innovative hypothesis based on the “gut microbiota‐iron death axis” also provides a new theoretical framework for flavonoids to improve OA progression by synergistically regulating the gut‐joint axis and inhibiting the ferroptosis cascade. In the future, we should focus on the systematic identification of flavonoid microbial metabolites with anti‐OA activity and their targets; to elucidate the molecular mechanism of specific flora‐metabolite‐host interaction, to explore the design strategy of flavonoid precursor drugs targeting intestinal flora, and to carry out multi‐omics integrated analysis, so as to fully reveal the network regulation mechanism of flavonoids in the treatment through the “gut‐joint axis” [131].

In summary, flavonoids, as a natural product with a wide range of sources and multiple effects, provide a promising new idea for the prevention and treatment of OA. Breaking through the bottleneck of bioavailability by targeted nano‐delivery technology and revealing new pharmacodynamic substances and targets by means of intestinal microbial metabolism research are expected to produce innovative OA therapeutic drugs or functional foods with efficacy, safety, and disease modification functions in the future.

## 5 Conclusion

The pathophysiology of OA is inflammatory, oxidative, leading to extracellular matrix breakdown, and eventually chondrocyte death. It is a multi‐faceted development of the network, which means that it is hard to hinder its development with one target intervention only. Over the past few years, research on the activity of flavonoids in vivo and in vitro has always demonstrated that they have demonstrated multi‐target and multi‐pathway therapeutic effects in the prevention and treatment of OA [132, 133]. It can simultaneously inhibit core inflammatory signals such as NF‐κB, MAPK, and NLRP3, and activate protective axes such as Nrf2/HO‐1, PI3K/Akt, and AMPK/SIRT1, thereby down‐regulating MMPs/ADAMTS expression, reducing chondrocyte apoptosis and pyroptosis, restoring autophagic flux, and improving subchondral bone remodeling [134, 135, 136].The core of the anti‐OA effect of flavonoids is that the subtle differences in their chemical structures determine the diversity of biological activities. The different modifications on the basic skeleton (C6─C3─C6) directly affect the antioxidant, anti‐inflammatory, and pharmacokinetic properties. The side chain structure of various subflavonoids is what defines their tissue distribution properties and targeting preferences: the antioxidant activity mainly depends on the B‐ring hydroxylation mode, such as quercetin, EGCG containing o‐diphenol or o‐triphenol hydroxyl groups, which is the key to efficient scavenging free radicals and chelating metal ions [45]. The anti‐inflammatory activity is related to the planar conformation of the molecule. The C2═C3 double bond of the C‐ring is conjugated with 4‐carbonyl (such as flavonoids and flavonols) to stabilize free radicals and inhibit pro‐inflammatory kinases such as MAPK. In terms of metabolism and targeting, methoxylation (such as nobiletin) can increase lipophilicity and metabolic stability, and improve oral bioavailability; although glycosylation (such as hesperidin) increases water solubility, it is often used as a prodrug and needs to be deglycosylated in vivo to release active aglycones [82]. The unique structural motif also gives target selectivity: isoflavones act on estrogen receptors by virtue of the 3‐phenyl structure; proanthocyanidins with high degree of polymerization enhance protein binding ability [92]. There are also differences in tissue distribution. For example, flavonols such as quercetin have a stronger inhibitory effect on the release of IL‐1β from synovial macrophages, while flavanones can break through the bottleneck of bioavailability through local targeted delivery [52].

Nevertheless, we must emphasize that these promising mechanisms are still mainly in the preclinical stage. The transformation from in vitro efficacy and animal efficacy to human clinical benefits is fraught with obstacles (poor oral bioavailability, extensive first‐pass metabolism, uncertain dose‐exposure‐response relationship, hepatotoxicity risk at pharmacological doses [97], and lack of rigorous clinical trial evidence for symptom improvement and structural disease modification). The future development of flavonoids in the field of OA should be guided by clinical transformation. Focus on breakthroughs in targeted nano‐delivery systems: develop hyaluronic acid‐functionalized nanoparticles, achieve active targeting of cartilage with the help of CD44 receptors, use the acidic environment of joints and high expression of matrix metalloproteinases to construct pH/enzyme dual‐responsive release, and use temperature‐sensitive gels to make injectable in‐situ reservoirs for sustained release for several weeks. Focusing on the metabolites of intestinal microflora, phenolic acid metabolites such as phenyl‐γ‐valerolactone were identified by large‐scale metabolomics and associated with OA outcomes [131, 137]. The co‐metabolic pathway of specific flora hydrolyzing glycosides and cracking into active phenolic acids was elucidated. Colon‐targeted preparations were designed to rely on flora metabolism to produce active substances to avoid the first‐pass effect, and a standardized metabolite biomarker panel was established and verified. A large‐scale RCT was carried out in the framework of precision medicine: a single compound with a purity of ≥95% and an HPLC fingerprint was used to explore the adaptive dose based on PopPK, uCTX‐II, COMP, and other markers to determine the minimum effective dose. MRI cartilage thickness/joint space width was the primary endpoint, WOMAC/KOOS and pain VAS were the secondary endpoints, and the patients were followed up for ≥24 months. At the same time, strict hepatotoxicity monitoring was carried out, and DSMB supervised and preset ALT>3 times the normal upper limit withdrawal rules [138], combined with electronic health records to establish real‐world evidence, a multicenter double‐blind placebo‐controlled phase IIb/III RCT was initiated.

In a word, flavonoids, as a kind of natural products with wide sources and multi‐directional effects, provide a promising way for the prevention and treatment of OA in theory. However, it should be considered as a candidate compound that requires extensive pharmaceutical optimization and rigorous clinical validation, rather than an established disease modification therapy.

## Author Contributions

**Haibin Wang**: data curation, resources, writing – original draft. **Shuqi Zheng**: data curation, methodology. **Chunlei Hua**: data curation, methodology. **Wanze Feng**: formal analysis, resources. **Zengxia Liu**: formal analysis, resources. **Yanhong Sun**: conceptualization, methodology, project administration, supervision, editing. **Minhui Li**: conceptualization, methodology, project administration, supervision, editing .

## Conflicts of Interest

The authors declare no conflicts of interest.

## Use of Generative AI and AI‐Assisted Technologies in the Writing Process

No AI tools were used to generate substantive scientific content, data, or conclusions. The authors take full responsibility for the accuracy and integrity of all content presented.

## Data Availability

No new data were generated or analyzed in support of this research.
